# Effect of chrysophanic acid on immune response and immune genes transcriptomic profile in *Catla catla* against *Aeromonas hydrophila*

**DOI:** 10.1038/s41598-020-79629-9

**Published:** 2021-01-12

**Authors:** Ramasamy Harikrishnan, Gunapathy Devi, Chellam Balasundaram, Hien Van Doan, Sanchai Jaturasitha, Einar Ringø, Caterina Faggio

**Affiliations:** 1 Department of Zoology, Pachaiyappa’s College for Men, Kanchipuram, Tamil Nadu 631 501 India; 2 Department of Zoology, Nehru Memorial College, Puthanampatti, Tamil Nadu 621 007 India; 3 Department of Herbal and Environmental Science, Tamil University, Thanjavur, Tamil Nadu 613 005 India; 4 Department of Animal and Aquatic Sciences, Faculty of Agriculture, Chiang Mai University, Chiang Mai, 50200 Thailand; 5 Science and Technology Research Institute, Chiang Mai University, 239 Huay Keaw Rd., Suthep, Muang, Chiang Mai, 50200 Thailand; 6 Norwegian College of Fishery Science, Faculty of Bioscience, Fisheries and Economics, UiT The Arctic University of Norway, Tromsø, Norway; 7 Department of Chemical, Biological, Pharmaceutical and Environmental Sciences, University of Messina, Piazza Pugliatti, Italy

**Keywords:** Immunology, Adaptive immunity, Gene regulation in immune cells, Innate immunity

## Abstract

The effect of chrysophanic acid (CA) (2, 4, and 8 mg kg^−1^) on the immunity and immune-related gene profile of *Catla catla* against *Aeromonas hydrophila* is reported. In both control and treated groups fed with 2 mg kg^−1^ (2 CA), the phagocytosis, hemolytic, myeloperoxidase content, and superoxide anion production decreased significantly between 6th and 8th weeks, whereas when fed with 4 mg kg^−1^ CA (4 CA) the H_2_O_2_ production and nitric oxide synthase increased significantly between 4th and 8th week. When fed with 2 CA and 4 CA diets, the total protein, bactericidal, and antibody titer increased significantly from the 4th week onwards. When fed with 2 CA, the IL-1β and IL-10 mRNA expression of head kidney leucocytes were significant between weeks 6 and 8. The expressions of toll-like receptors significantly increased when fed with a 4 CA diet from 4th week onwards. The 4 CA group significantly increased in TNF-α, TNF receptor-associated factor 6 (NOD), which influences protein expression, after the 4th week. The mRNA transcription of MHCI, lysozyme-chicken and goose type expressions significantly increased in 4 CA group within the 4th week. In summary, the dietary administration of 4 mg kg^−1^ of CA (4 CA) provides better immunity and enhances the up-regulation of immune-related genes in *Catla* against *A. hydrophila*.

## Introduction

Today’s growing world population has led to increasing demand for aquaculture as a luxury and cheap protein source^1^. Correspondingly, the current aquaculture practice has shifted from extensive to semi- or intensive systems. In the year 2016, the global aquatic food production has exceeded 171 million tons^2^. Fish production in the first two quarters of 2017 and 2018 increased to 5.80 million tons^3^*.* Among the Indian major carps (IMCs) *Catla catla* is the most commonly farmed freshwater fish due to its size, good flavor, high protein content, omega-3 fatty acids, yet with fewer triglycerides, which promote brain function^4^; besides species like *C. catla* are also a cheap source of aqua-protein (about 2 US dollars/kg in countries like India). However, an intensive aquaculture system triggers a highly stressful environment that adversely affects the immune system, making the cultivated fish more vulnerable to infectious agents^5^. Besides, any culture system with maximum rearing density triggers frequent outbreaks of several infectious diseases, increasing the host susceptibility, virulence of the pathogen, and health-related problems^6–8^. Like other IMCs, *Catla* suffers from several infections, including aeromoniasis, edwardsiellosis, and epizootic ulcerative syndrome (EUS)^9^. Among these, *Aeromonas hydrophila* is a leading bacterial pathogen known to cause symptoms like haemorrhagic septicaemia, infectious dropsy, ulcerative lesion, and fin rot resulting in mass mortality^10,11^ affecting the quality and quantity of the size of harvest significantly. To manage these diseases, fish farmers conventionally use broad-spectrum antibiotics and chemotherapeutics, which often lead to frequent outbreaks and spread of resistant strains and environmental threats, creating further problems^12,13^. Vaccines are an effective prophylactic measure in aqua-practice to inhibit or control of infectious diseases; however, its success rate varies since, as they are pathogen specific^14^. Furthermore, the vaccines developed for intracellular fish pathogens are yet to become successful^15,16^.

In this regard, natural immunostimulants have become an attractive alternative. In fish disease management, they are widely accepted to sustain aquaculture, as they are biocompatible, biodegradable, often readily available, eco-friendly, and safe for human health^17,18^. Several immune-stimulants are known to afford disease protection by boosting non-specific and specific immune systems^19–22^. The use of herbals and their bioactive constituents can circumvent the use of traditional chemotherapies. In plants, polyphenols like anthraquinones, lignans, flavonoids, and aromatic acids are widely distributed; they exhibit antioxidant properties^23^; in human health management, the herbal medicine also play a progressive role in controlling cardiac disease^24^, cancer^25^, and viral infection^26^. Chrysophanic acid or chrysophanol (1, 8-dihydroxy-3-methyl-anthraquinone) that come under the anthraquinone family is widely distributed in Chinese herbs (*Rheum officinale* and *Polygpnum cuspidatum*), and exhibit anti-bacterial and anti-fungal properties^27,28^. Chrysophanic acid (CA) induces reactive oxygen species (ROS) production, dysfunction of mitochondria, and damage of ATP and DNA, causing necrotic cell death in human liver J5 cancer cells^29–31^. CA is also known to stimulate cytosolic Ca21 production, and cause a decrease in Dilated Cardiomyopathy (DCM) levels^32^. However, no detailed research has been conducted on CA’s effect with reference to immunity and cytokine gene modulations in aquatic species. This work aims to find out the effect of diets containing CA on the innate and adaptive immunity and expression of immune-related gene expression in *C. catla* against *A. hydrophila*.

## Results

### Immunological response

**Figure 1:**
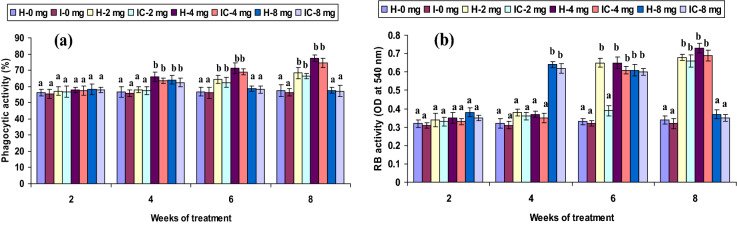
(**a**) Phagocytic activity and (**b**) respiratory burst (RB) activity of catla (n = 6) head kidney leucocytes against *A. hydrophila*. *H* healthy, *I* infected. Data are expressed as mean ± SD and the statistically significant difference (*P* < 0.05) between mean values as indicated in different letters in each column.

**Figure 2:**
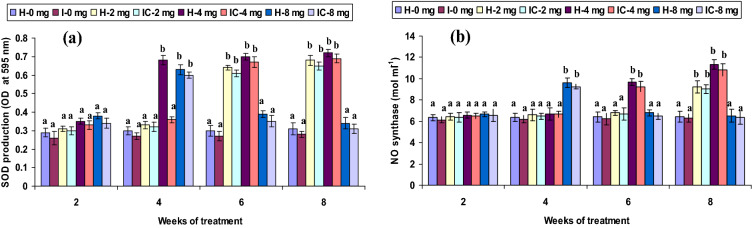
(**a**) Superoxide anion (SOD) radical production and (**b**) nitric oxide (NO) synthase of catla (n = 6) head kidney leucocytes against *A. hydrophila*. *H* healthy, *I* infected. Data are expressed as mean ± SD and the statistically significant difference (*P* < 0.05) between mean values as indicated in different letters in each column.

**Figure 3:**
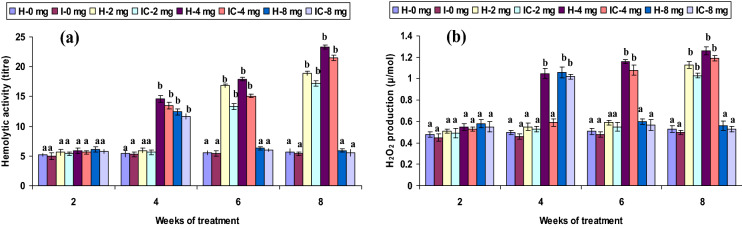
(**a**) Hemolytic activity and (**b**) hydrogen peroxide (H_2_O_2_) production of catla (n = 6) head kidney leucocytes against *A. hydrophila*. *H* healthy, *I* infected. Data are expressed as mean ± SD and the statistically significant difference (*P* < 0.05) between mean values as indicated in different letters in each column.

**Figure 4:**
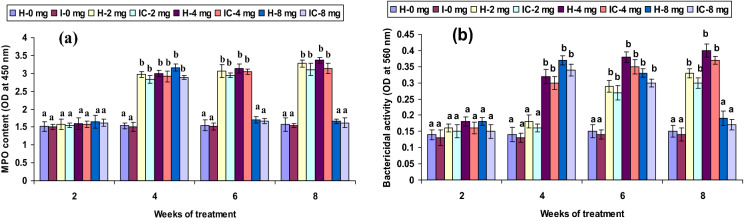
(**a**) Myeloperoxidase (MPO) content and (**b**) bactericidal activity of catla (n = 6) head kidney leucocytes against *A. hydrophila*. *H* healthy, *I* infected. Data are expressed as mean ± SD and the statistically significant difference (*P* < 0.05) between mean values as indicated in different letters in each column.

**Figure 5:**
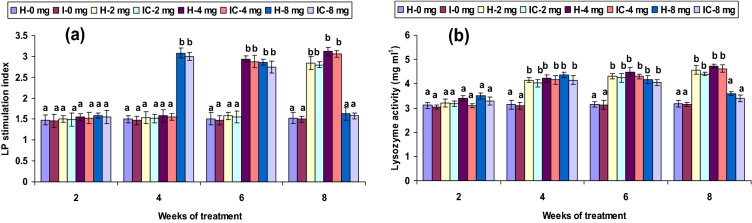
(**a**) Lymphocyte proliferate (LP) stimulate index and (**b**) lysozyme activity of catla (n = 6) head kidney leucocytes against *A. hydrophila*. *H* healthy, *I* infected. Data are expressed as mean ± SD and the statistically significant difference (*P* < 0.05) between mean values as indicated in different letters in each column.

**Figure 6:**
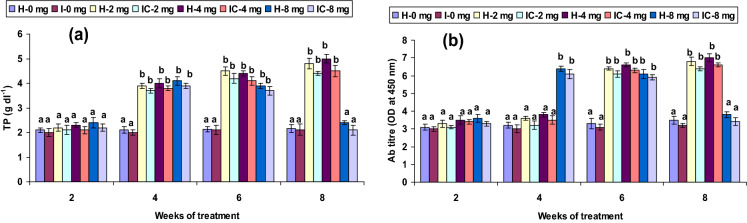
(**a**) Total protein (TP) level and (**b**) antibody (Ab) titre of catla (n = 6) head kidney leucocytes against *A. hydrophila*. *H* healthy, *I* infected. Data are expressed as mean ± SD and the statistically significant difference (*P* < 0.05) between mean values as indicated in different letters in each column.

Both healthy and infected groups fed with the 2 CA enriched diet had increased phagocytic and hemolytic activity and SOD generation (*P* < 0.05) between the 6th and 8th weeks, but not in the 2nd or 4th week. Fish fed with 4 CA diet in both groups exhibited these activities at the 4th, 6th, and 8th weeks. However, the 8 CA administration significantly enhanced these parameters in the 2nd week only; no differences were observed in either the 1st week or after the 6th week (Figs. 1a, 2a, 3a). In both 2 CA groups, enhanced LP index, MPO content, bactericidal action, and antibody levels were observed between the 6th and 8th weeks. A similar trend existed in both groups fed with 4 CA from week 2 to 8. The maximum values of the immune parameters were obtained when both groups were fed the 8 CA diets between weeks 2 and 4; however, no increases were observed in the initial study period (week 1) or after prolonged periods (after week 8) (Figs. 4a,b, 5a, 6b).

Low respiratory burst (RB) activity was observed in both 2 CA and 4 CA groups in the 2nd and 4th weeks; however, the highest (*P* < 0.05) values were noted after week 6. In both 8 CA groups, the highest RB activity (*P* < 0.05) manifested between weeks 4 and 6, but these values were insignificant in the 1st week and after week 8 (Fig. 1b). The nitric oxide (NO) synthase was low in both 2 CA groups (weeks 2–6); increased significantly after the 8th week. Similarly, both 4 CA groups had slightly higher levels (*P* > 0.05) of NO synthase in the 2nd and 4th weeks, and significantly (*P* < 0.05) higher levels between weeks 6 and 8. NO synthase levels varied moderately (*P* > 0.05), except in week 2 (Fig. 2b).

Hydrogen peroxide (H_2_O_2_) production did not differ (*P* > 0.05) significantly in the 2 CA fed groups from weeks 2 to 6 but attained significant levels (*P* < 0.05) in the 8th week. The highest H_2_O_2_ production was observed in both 4 CA groups between the 4th and 8th weeks, and the lowest production activity (*P* > 0.05) occurred in the 8 CA groups (Fig. 3b). Lysozyme levels and TP levels decreased slightly (*P* > 0.05) in the 2nd week in the 2 CA and 4 CA groups. These values rose significantly (*P* < 0.05) in both 8 CA groups from 4 to 8 weeks, though increases did not vary in either the 1st or after the 8th weeks (Figs. 5b, 6a).

### Immune gene expression

**Figure 7:**
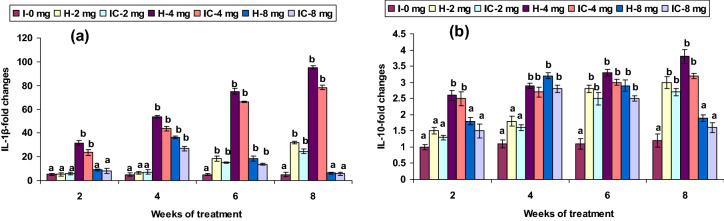
Expression pattern of (**a**) IL-1β and (**b**) IL-10 gene relative to β-actin in the head kidney of catla (n = 3) against *A. hydrophila*. *H* healthy, *I* infected. Values are expressed as mean ± SD and the statistical difference between means (*P* < 0.05) indicated in different letters in each column.

**Figure 8:**
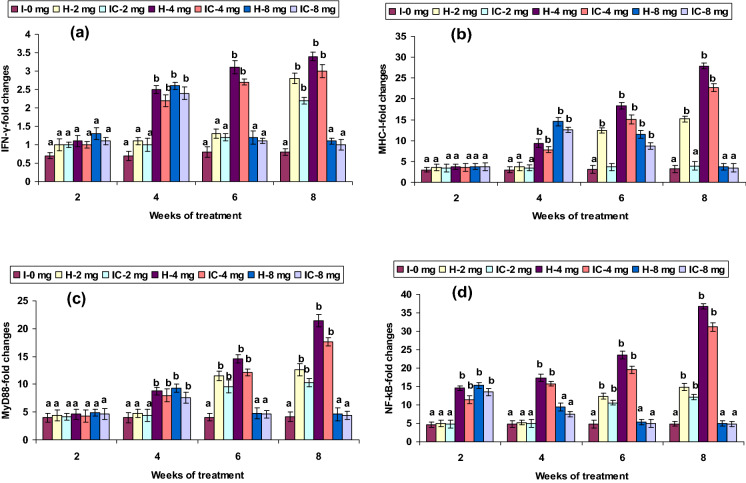
Expression pattern of (**a**) IFN-γ, (**b**) MHC-I, (**c**) MyD88, and (**d**) NF-kB relative to β-actin in the head kidney of catla (n = 3) against *A. hydrophila*. *H* healthy, *I* infected. Values are expressed as mean ± SD and the statistical difference between means (*P* < 0.05) indicated in different letters in each column.

**Figure 9:**
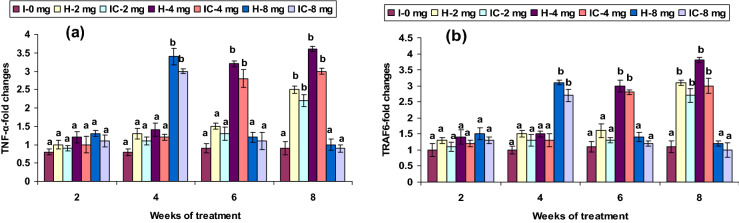
Expression pattern of (**a**) TNF-α and (**b**) TRAF6 relative to β-actin in the head kidney of catla (n = 3) against *A. hydrophila*. *H* healthy, *I* infected. Values are expressed as mean ± SD and the statistical difference between means (*P* < 0.05) indicated in different letters in each column.

**Figure 10:**
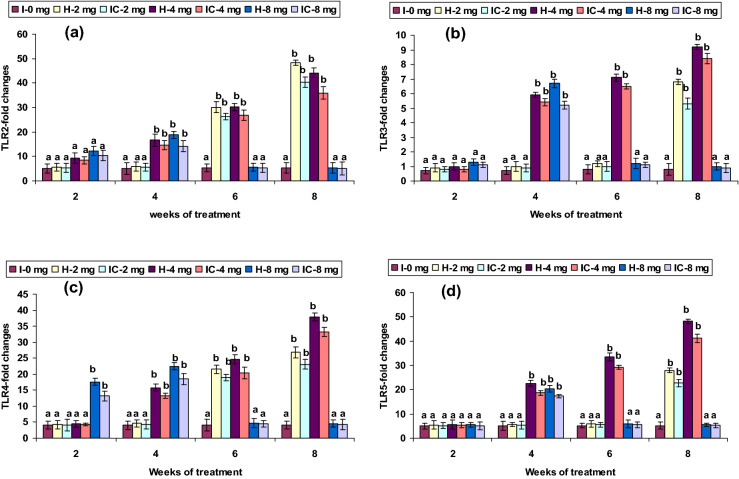
Expression pattern of (**a**) TLR-2, (**b**) TLR-3, (**c**) TLR-4, and (**d**) TLR-5 relative to β-actin in the head kidney of catla (n = 3) against *A. hydrophila*. *H* healthy, *I* infected. Values are expressed as mean ± SD and the statistical difference between means (*P* < 0.05) indicated in different letters in each column.

The mRNA transcripts of IL-1β and 10, TLR-4, as well as MHC-I in head kidney leucocytes, were significantly low between weeks 2 and 4 when fed with 2 CA in both groups, whereas its expression was up-regulated between the 6th and 8th weeks. Feeding with 4 CA could induced IL-1β, IL-10, TLR-4, and MHC-I mRNA expression in both groups significantly from weeks 4 to 8, compared with that of the control. However, the IL-1β, IL-10, TLR-4, and MHC-I mRNA expression were significant in both groups in the 4th and 6th weeks in the 8 CA groups; yet no up-regulation was observed in the first or after the 8th week (Figs. 7a,b, 8b, 10c). The expression levels of TRAF6 and TNF-α were not significantly up-regulated in the 2 CA groups from week 2 to 6, while significant up-regulation was observed in the 8th week. Both groups fed with 4 CA showed down-regulation in the 2nd and 4th weeks, whereas up-regulation was observed in the 6th and 8th weeks. Nevertheless, the TRAF6 and TNF-α expression levels were slightly higher in the groups fed with 8 CA from week 4 to 8 (Fig. 9a,b).

**Figure 11:**
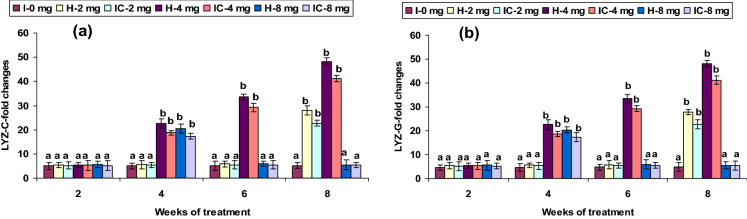
Expression pattern of (**a**) LYZ-C and (**b**) LYZ-G relative to β-actin in the head kidney of catla (n = 3) against *A. hydrophila*. *H* healthy, *I* infected. Values are expressed as mean ± SD and the statistical difference between means (*P* < 0.05) indicated in different letters in each column.

**Figure 12:**
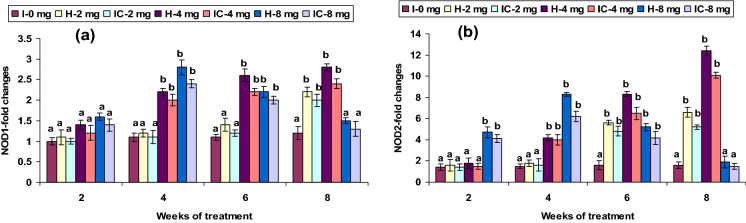
Expression pattern of (**a**) NOD1 and (**b**) NOD2 relative to β-actin in the head kidney of catla (n = 3) against *A. hydrophila*. *H* healthy, *I* infected. Values are expressed as mean ± SD and the statistical difference between means (*P* < 0.05) indicated in different letters in each column.

The expression of INF-γ, TLR3, TLR5, Lyz-C, and Lyz-G genes showed no up-regulation in both 2 CA groups from weeks 2 to 6; however, mRNA gene expressions were up-regulated in the 8th week. The 4 CA groups had a significant induction of INF-γ, MyD88, TLR3, TLR5, Lyz-C, and Lyz-G genes expression between weeks 4 and 8, but not in the 1st week (Figs. 8a,c, 10b,d, 11a,b). Interestingly, a slight up-regulation of NF-kB and TLR2 expression was observed in weeks 2 and 4 in the 2 CA group. However, there was a significant up-regulation between weeks 6 and 8. Similar results were found with the 4 CA and 8 CA groups in the 2nd and 4th weeks; however, all the values decreased after the 6th week (Figs. 8d, 10a). The expression of NOD1 and NOD2 were not significantly improved in the 2 CA group (weeks 2–6), yet there was a sudden up-regulation in the 8th week. Significant NOD1 and NOD2 mRNA expressions were also observed in the 4 CA group after the 4th week, as well as in the 8 CA groups (weeks 4 and 6), but not in the 1st week or after the 8th week (Fig. 12a,b).

### RPS

**Figure 13:**
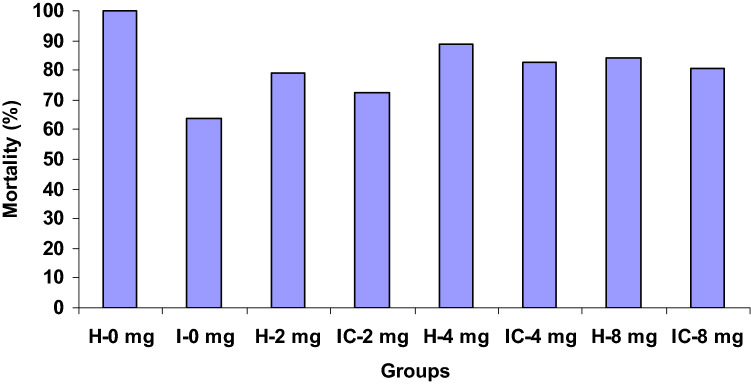
Survival rate in *C. catla* post-challenged and un-challenged *A. hydrophila* groups treated with different concentration of (0 mg, 2 mg, 4 mg, 8 mg) of chrysophanic acid (CA) observed at the end of experiment. *H* healthy, *I* infected.

Survival was 100% in group I (H-0 mg). In both post-challenged and un-challenged groups (groups III and IV), treated with 4 CA and 8 CA resulted in higher survival rates between 80.5 and 89%. On the other hand, post-challenged or un-challenged (groups II and VI) treated with 2 CA had low survival between 72.5 and 79%. However, post-challenged group V treated with the control diet had the least survival of 64% (Fig. 13).

## Discussion

The ability of phagocytic cells to kill the invading pathogens is an essential innate defense mechanism^33^. In the present study, phagocytic activity significantly improved in both groups (*P* < 0.05) with doses of 2 mg kg^−1^ CA (2 CA) between weeks 6 and 8; and in the 4 CA group between weeks 4 and 8. The phagocytic leucocytes harvested from HK of the 2 CA and 4 CA groups revealed high RB activity, attributed as a metabolic function due to CA in the 6th and 8th weeks, resulting in higher amounts of SOD synthesis. The HK leukocytes RB activity is another important innate immune function widely considered a bio-indicator of immune-competence, exclusively triggered by immunostimulants^33,34^. Extracellular O_2_^−^ production was highly significant in the 2 CA groups (6 and 8 weeks) and the 4 CA groups (4–8 weeks). Similar results were found in rohu, in which a higher level of O_2_^−^ production was reported (Days 4, 6, and 14) post-vaccination with *A. hydrophila*^35^. The O_2_^−^ production in macrophages exhibits immunity after the initiation of phagocytes in fish that generate reactive oxygen products; such as H_2_O_2_ and OH, during a period of robust O_2_ intake, termed as RB^36^. This ability to kill microorganisms by phagocytes has an essential role in anti-microbicidal mechanisms in fish^37^. The trout HK leucocytes collected after zymosan exposure showed maximum oxygen consumption or RB activity that produced 12 nM or 13 nM superoxide anion for 10^7^ cells^38^. In the present study, both groups fed 2 and 4 mg kg^−1^ CA diets (2 CA and 4 CA) showed no significant change in RB activity in weeks 4–6. These results were in agreement with a recent study in which a significant observance of RB activity was reported in Atlantic salmon and rainbow trout against *Lepeophtheirus salmonis*^39^. However, significantly high RB activity was observed in *Pangasianodon hypophthalmus* against pathogen^40^. Similarly, Munoz, et al.^41^ reported that a higher O_2_^−^ level afforded protection to *Dicentrarchus labrax* against *Sphaerospora dicentrarchi*. Natural immunostimulants do not significantly affect the innate defense response in fish^42,43^; numerous studies have reported a higher RB activity in fish through various immunostimulants^44,45^. H_2_O_2_ production was significantly high in the 8th week of the 4 CA feeding, whereas the RB activity in the 8 CA group was manifested in the 2nd week. Similar increases in H_2_O_2_ production were observed in *Psetta maxima* and *Sparus aurata* via the administration of glucan^46^. High ROS production due to glucan may be associated with augmented bactericidal and phagocytosis, as reported in Atlantic salmon and rainbow trout^47,48^. The ability to generate H_2_O_2_ was reported in neutrophils due to O_2_ breakdown during RB in channel catfish^49^. However, in the present study, the H_2_O_2_ production was low in both groups fed 2 mg kg^−1^ CA (2 CA) from week 2 to 6. Similarly, the native or heat-denatured lectin of *Abrus precatorius* induced low phagocytosis levels, bactericidal, and H_2_O_2_ production in mice macrophages^50^.

A significantly higher proliferative response was observed in both 2 CA groups after week 6, while in the 4 CA group, the same effect was observed after week 4. Consequently, with the administration of the *A. hydrophila* vaccine, the proliferative response in fish increased^51^. A lower response was observed in the earlier stage, week 2, in the 2 CA group, suggesting the induction of low memory. However, the potential influence of leukocyte function on enhancing specific immunity related to immunostimulant is yet to be explored. Notably, NO production was directly related to the granulocytes associated with the innate immune system^52^. In this study, NO synthase production was low in both 2 CA groups between weeks 2 and 6, which became significant in the 8th week. However, the 4 CA groups produced a significantly higher level of NO synthase in weeks 6 and 8. Das et al.^35^ reported similar effects of NO production in leukocytes in rohu against *A. hydrophila*. Consequently, the present results suggest that CA potentially influences NO synthesis of ROS and RNS in stimulated leukocytes as an immediate response to pathogens.

Neutrophil granules release MPO enzymes during oxidative RB that produce toxic hypochlorous acids that react with pathogenic microorganisms^53^. MPO content was highly significant (*P* < 0.05) in both 2 CA groups at 6 and 8 weeks, similar to that of the 4 CA groups from weeks 2 to 8. A significantly elevated level of MPO content was reported in *P. hypophthalmus* against monogenean infection^40^. Conversely, a recent study in rohu found that MPO activity was not significantly different between healthy and infected fish^54^. The present results indicate that a relatively lower concentration (2 mg kg^−1^ CA) stimulates MPO after 6 weeks, whereas a medium concentration (4 mg kg^−1^ CA) stimulated MPO earlier, at week 4. Hemolytic activity was statistically influenced (*P* < 0.05) in the 2 CA groups in weeks 6 and 8, whereas the 4 CA groups induced MPO between weeks 4 and 8. Among various immune mechanisms, only the complement pathway has the potential to avert microbial infection. Therefore, the hemolytic exertion in fish serum is recognized as an intersperse complement pathway, which plays a vital role against infectious pathogens^55^. In this study, a significant hemolysin titer was obtained with the 4 mg kg^−1^ CA, suggesting the influence of complement pathways against pathogens^56^. However, the groups fed with a low dose (2 mg kg^−1^ CA) produced a high hemolysin titre in the later stages, whereas a high dose (8 mg kg^−1^ CA) produced similar results in the earlier stages. Additionally, a more recent study reported less hemolysin titre in rohu against dactylogyrid monogenean^54^. However, these changes may be dependent upon fish species, the pathogenicity of the microbes, or the blocking of certain microbe epitopes, capable of influencing the alternate complement cascade.

Apart from the RB mechanism, lysozyme is a lysis enzyme produced by granulocytes during non-specific oxygen-independent pathways that play an essential role in innate immunity^57–59^. In vertebrates, lysozyme is an imperative humoral component in the systemic and mucosal immune systems; it further acts as a defensive factor against pathogenic microorganisms^57^; lysozyme secreted by human granulocytes attach to the hyphae of *Candida albicans*^60^. These studies strongly suggest the existence of a lysozyme-conflict defence mechanism in fish against pathogens. TP is also an important compound involved in the immune system. Both lysozyme activity and TP concentration reached significant levels (*P* < 0.05) in both groups fed with 2 CA and 4 CA diets between the 4th and 8th weeks. Elevated serum lysozyme activity was also reported in the immunostimulant administration in rainbow trout and rohu^61,62^.

Bactericidal and antibody levels were statistically improved (*P* < 0.05) in both 2 CA groups after week 6 and in the 4 CA groups from weeks 4 to 8. The relationship between antibody production and protection against pathogens has been demonstrated in *Catla* against furunculosis^51^. However, the significance of protection or resistance to pathogen within antibodies further suggests cellular immunity following immunostimulants. Antibody synthesis depends primarily on effector-cell proliferation and its specific antibody secretion or memory cell differentiation, which representing the complex progression needed for cytokine secretion and cell cooperation^63^.

In the present study, immune defense system changes when infected with *A. hydrophila* led to both up- and down-regulation of selected immune genes. The immune-related gene expression patterns in fish have been investigated only recently; and, consequently, the understanding of immunity to pathogen is limited. IL-1β is an important inflammatory mediator in microbial infections^64^. In the 2 mg kg^−1^ CA diets, the IL-1β and IL-10 expression levels in HK were significantly low between weeks 2 and 4, and relatively high in weeks 6 and 8. The gene’s significant up-regulation was also present in the 4 CA groups in the 6th and 8th weeks. IL-1β was up-regulated in the skin of trout against *Gyrodactylus derjavini*^65^. However, in the present study, IL-1β showed a significant down-regulation in *Catla* infected with *A. hydtophila*; suggesting the release of pathogen toxins in different phases of pathogen attachment, which play a significant role in arbitrating inflammation, thereby leading to down-regulation^62^; an increase in IL-1β and IL-10 expression. The exact actions of CA involvement in fish against *A. hydtophila* may be contained within the immune system. IL-10 is involved in the synthesis of the multifunctional cytokine inhibitory factor, which promotes immune-suppressive function^66^, and plays a major role in undermining the proinflammatory cytokines responses; such as IL-1β, TNFα, and IFN-γ; as well as preventing tissue damage^67,68^. Increased IL-10 mRNA expression levels have been reported in fish due to different immunostimulants related to the transcription arising in innate immune cells, such as macrophages^69,70^.

TLRs facilitate inflammatory immune responses when binding to molecules with PAMPs, and trigger an inflammation-related genes expression through signal transduction^71,72^, also referred to as endogenous TLR ligands, which acts as an ominous early alarm to innate and adaptive immunities^73^. The TLR2, TLR3, TLR4, and TLR5 expressions were significantly up-regulated in the 4 CA groups after 4 weeks and in the very early and late stages in the 2 CA and 8 CA groups. Modulation of negating innate immune gene receptors, like TLRs and NLRs, plays a fundamental role in forming an Ig repository^74^. A recent study reported that similar functions were demonstrated in the embryo stages in different organs or tissues of *L. rohita* TLR2 and TLR5 genes^75^. The TLR4 has been recently demonstrated with the ability to recognize viral or bacterial motifs^76^.

TRAF6 and TNF-α were up-regulation in the HK in both 4 CA groups after the 6th week and after the 8th week in the 2 CA groups. A similar expression pattern of TNF-α was recorded in zebrafish embryos and adults against *Edwardsiella tarda* infection^77^ and in carp HK, due to LPS^78^. However, TRAF6 and TNF-α expression presented low in the HK in both 4 CA groups between the 2nd and 4th weeks and between the 4th and 6th weeks in the 8 CA groups. Similarly, a low TNF-α expression was reported in rohu HK against *E. tarda*^79^. The TRAF6 response to different PAMPs treatments in *Epinephelus tauvina* revealed its contribution in influencing immune responses^80^. MHC receptors are associated with antigen-presenting cells (APCs), which assist T-cells in initiating the immune system^81^. In HK, leucocyte MHC-I expression was up-regulated in the 2 CA groups in the 6th and 8th weeks and the 4 CA groups on or after the 4th week. The high level of MHC expression in *Catla*, due to *A. hydrophila*, suggests that MHC contains cells (macrophages) within the HK that mediate the inflammation. However, no such change in MHC-I expression was observed in the 2 CA groups between weeks 2 and 4. Gharbi et al.^82^ reported the MHC receptors susceptibility in Atlantic salmon to *L. salmonis*.

The up-regulation of NF-kB occurred in both 2 mg kg^−1^ CA groups in weeks 6 and 8, whereas MyD88 expression significantly was up-regulated in the same period, in the 4 CA groups in weeks 4–8, and the 8 CA in weeks 4–6. The IL-10 mechanism was described in *Catla* by blocking the NF-kB-signals in the HK. Association of PAMP-TLR induced oligomerization, triggered intracellular signaling cascade via recruitment of the myeloid differentiation factor 88 (MyD88)-dependent or -independent pathways^83^. Combined stimulation of intracellular MAP kinase pathway and Iκβ deprivation irritates the triggering of transcription factors, like AP-1 and NF-κB; which triggers proinflammatory cytokine secretion in the B and T cells leading to B cell proliferation^84^. In zebrafish, the TLR4-induced MyD88 dependent signals triggered the regulation of anti-inflammation^85^. The results herein suggest that both MyD88-dependent and independent TLR-induced signaling pathways together to regulate the activation of *catla* lymphocytes and lymphoid organs. NF-κB is capable of binding to Ig kappa light-chain of pathogens encountered within the B cells^86^. Therefore, the tight NF-κB phosphorylation and adhesion regulation are necessary to inhibit dysfunction of immune function^87^. Thus, the sudden increase in NF-κB levels observed in this study indicates their vital role in the pathophysiology linked to TLR signaling.

When fed with 4 mg kg^−1^ CA, both NOD1 and NOD2 demonstrated significant expression on or after week 4. In addition to TLRs, NLRs is also responded to microbial components^88^ and endogenous ligands, as a result of tissue or cellular injuries^89^. The TLR2 and TLR5, NOD1, and NOD2 genes were up-regulated carp’s embryonic stages^90,91^. The TLR and NOD constitutive expression suggest the possibility of innate immune receptors in the early stage of CA treatment in *Catla*.

The chicken (Lyz-C) and goose (Lyz-G) gene expressions were not up-regulated in the 2 CA groups in the 8th week and in the 4 CA groups between weeks 4 and 8. Similar Lyz-C and Lyz-G up-regulation was recorded in Japanese flounder kidney, intestine, heart, whole blood, spleen, and ovary against *E. tarda*^92,93^. Hence, the Lyz gene up-regulation may be responsible for the rise in serum lysozyme concentration in *Catla* fed with CA diets against *A. hydrophila* infection.

There was 100% survival in H-0 mg, but only 64% of survival rate was observed in the infected group fed with the control diet. Both post-challenged and un-challenged groups treated with median dose (4 mg kg^−1^) and high doses (8 mg kg^−1^) of CA had high survival (between 80.5 and 89%), but the low survival rate was observed in both groups treated with low dose (2 mg kg^−1^) of CA resulting in low survival (between 72.5 and 79%). Similar reports in different fishes when fed with diets enriched with various active constituents indicate the same trends^94–96^.

In conclusion, to the best of our knowledge, this is the first report on the positive influence of CA in *C. catla* on innate and adaptive immune responses. The optimum level of CA is 4 mg kg^−1^, which manifested during the 2nd week of treatment. The pathway related to the inflammation and immunomodulation of genes also triggered a similar response. The 8 mg kg^−1^ of CA resulted in such response much later, after the 6th week. Further comprehensive investigations are necessary to explain immune gene expressions’ ability to elucidate the mechanism of action in other fish species, with different chrysophanol doses, against different pathogens.

## Materials and methods

### Formulation of experimental diet

The basal/control diet contained maize grain, fish meal, finger millet, and pearl millet as protein sources; rice-bran and wheat-flour as carbohydrate sources; and groundnut oil cake and vegetable oil as lipid sources (Table 1). Each ingredient was finely pounded and mixed with the required water volume to make a soft bread. The prepared feed was kept in an aluminum vessel and steamed in a pressure cooker for 15 min (at 15 psi). The formulated blend was cooled at room temperature (RT); then incorporated with the pre-mix of vitamins and minerals. Four experimental diets were prepared, by mixing chrysophanic acid (CA) in four different concentrations: (1) 0 mg kg^−1^, (2) 2 mg kg^−1^ (2CA), (3) 4 mg kg^−1^ (4CA), and (4) 8 mg kg^−1^ (8CA). Pellet feeds were prepared using a manual pelletizer (2 mm). The prepared diets were immediately oven-dried at 60 °C for 12 h. and tightly packed, stored in appropriate containers, and labeled. The experimental pellet feed constitutions were analyzed using regular procedures.

**Table 1:** Ingredients on dry matter basis and proximate composition experimental feed used in this study.

Ingredients	Chrysophanol			
	0 mg	5 mg	10 mg	15 mg
Maize grain	10.000	10.000	10.000	10.000
Fish meal	10.000	10.000	10.000	10.000
Finger millet	10.000	10.000	10.000	10.000
Pearl millet	10.000	10.000	10.000	10.000
Rice bran	25.000	24.992	24.996	25.992
Wheat flour	10.000	10.000	10.000	10.000
Groundnut oil cake	20.000	20.000	20.000	20.000
Vegetable oil	2.000	2.000	2.000	2.000
Vitamin + mineral mix^a^	2.000	2.000	2.000	2.000
Common salt	1.000	1.000	1.000	1.000
Chrysophanic acid	0.000	0.002	0.004	0.008
**Proximate composition (dry matter basis, g kg**^−**1**^**)**				
Crude protein	38.96	38.22	38.45	37.92
Crude lipid	11.36	11.27	11.08	10.89
Crude fiber	2.57	2.38	2. 23	2.02
Ash	9.33	9.26	9.18	9.04
Moisture	6.63	6.46	6.31	6.15

^a^Vitamin and minerals pre mix: Vitamin A: 700,000 IU, Vitamin D3: 140,000 IU, Vitamin E: 500 mg, Vitamin B12: 1000 mcg, Folic acid: 100 mg, Nicotinamide: 1000 mg, Copper: 1200 mg, Cobalt: 150 mg, Iron: 1500 mg, Zinc: 3000 mg, Iodine: 325 mg, Selenium: 10 mg, Magnesium: 6000 mg, Manganese: 1500 mg, Potassium: 100 mg, Calcium: 27 mg, Phosphorus: 13 mg, Sulphur: 0.72 mg, Fluorine: 300 mg.

### Aeromonas hydrophila

*Aeromonas hydrophila* (MTCC 1739) was acquired at Himedia (India), and sub-cultured at 37 °C for 24 h. in a nutrient broth. The bacterial suspension obtained was centrifuged at 3000 × *g* for 10 min, and the supernatant was then discarded. The remaining pellet bacteria was re-dissolved in phosphate buffered saline (PBS, pH 7.4); until an optical density (OD) of the suspension [0.5 at 456 nm at 1 × 10^6^ colony forming unit (CFU)] was achieved using a microplate reader and stored in a deep freezer for further use.

### Fish

Healthy, *Catla catla* (36.7 ± 2.1 g, 20.3 ± 2.9 cm) were procured in a local farm and kept in 500 L fiber-reinforced plastic (FRP) containers, sufficiently aerated, and filtered with de-chlorinated freshwater. The fish were immediately immersion in a KMnO_4_ solution for 2 min to evade any dermal infection and then kept for 2 weeks under standard laboratory conditions within natural photoperiod. During the acclimation period, fish were provided the control diet (Table 1). Fecal and unfed materials were siphoned off daily to avoid the accumulation of ammonia content in the tanks. The following measurements were recorded: pH 7.2 ± 0.05, dissolved oxygen 8.8 ± 0.02 mg l^−1^, temperature 24 ± 1 °C, and CaCO_3_ 190 ± 0.2 ppm.

### Experimental setup

Fish were arbitrarily strewn into eight groups of 25 fish (8 × 25 = 200 fish), in three replicates (3 × 200 = 600 fish): Group I, healthy (non-challenged) fish fed with the basal control diet (0 mg kg^−1^) CA [0CA or H-0 mg]; Group II, the healthy fish fed the dietary inclusion of 2 mg kg^−1^ [2CA or H-2 mg], Group III: fed the 4 mg kg^−1^ [4CA or H-4 mg] CA diet, Group IV: fed the 8 mg kg^−1^ [8CA or H-8 mg] CA diet; Group V, infected (or challenged) fish fed the basal control diet (0 mg kg^−1^) CA [0CA or I-0 mg]; and the challenged infected fish fed dietary inclusion of 2 mg kg^−1^ [2CA or IC-2 mg], Group VI; 4 mg kg^−1^ [4CA or IC-4 mg], Group VII; and 8 mg kg^−1^ [8CA or IC-8 mg] CA, Group VIII. Groups I through IV represented non-infected or non-challenge fish, injected with 0.5 mL PBS; whereas groups V through VIII contained fish challenged with 0.5 mL PBS containing *A. hydrophila* at 1 × 10^6^ CFU. The specified diets were provided twice daily at 10:00 and 17:00 throughout the experiment.

### Blood and tissues sampling procedure

Six fish from each group were arbitrarily chosen at the end of weeks 2, 4, 6, and 8 post-challenged with *A. hydrophila*. Post-anesthetized (MS-222, Sigma, USA), blood samples were drawn separately from the cardinal vein via a 1 mL plastic syringe. Each blood sample was equally divided into two separate sterile tubes with and without heparin, respectively. The heparinized blood was immediately analysed for hemato-biochemical analysis and immunological study, whereas the non-heparinized blood samples were permitted to clot at RT, then preserved at 4 °C for 4 h. All samples were centrifuged (2300 × *g*) for 5 min at 4 °C. The resulting serum was collected and stored in individual sterile tubes (− 80 °C) for further analyses. Lastly, the anterior kidney was dissected out aseptically from each fish, and RNAlater was added (Ambion, USA) and then preserved at − 80 °C awaiting RNS extraction.

### Immunological assays

#### Non-specific immune parameter

Phagocytosis was analysed via *Aeromonas hydrophila*, whereas the respiratory bursts were determined by the reduction of nitroblue tetrazolium (NBT) assay (Sigma, MO, USA) to measure neutrophils reactive oxygen radical production^97^. Bactericidal activity was determined using a 96-well microtiter plate with 3-(4, 5 dimethyl-2-yl)-2, 5-diphenyl tetrazolium bromide (MTT), according to the method of Kampen et al.^98^. Serum lysozyme levels were measured via turbidimetric assay Ellis^57^. Nitric oxide synthase (NOS) was analysed as prescribed by Lee et al.^99^. The production of hydrogen peroxide (H_2_O_2_) was measured by phenol red oxidation method using horseradish peroxidase^33^. The HK leukocytes proliferative reactions were measured via MTT assay^100,101^. The synthesis of superoxide anion (SOD) was estimated following^51^. Respiratory burst (RB) in terms of oxygen radical generation was measured by phagocytes using NBT reduction assay^97^. Total serum myeloperoxidase (MPO) and total protein contents were measured by the technique employed by Mohanty and Sahoo^79^ and Gornall et al.^102^. Serum hemolytic activity was estimated by microtitre plate^54^.

#### Specific immune parameters

The serum antibody levels of fish infected with *A. hydrophila* was determined using an indirect enzyme linked immunosorbant assay (ELISA)^103^ with some modifications^104^.

### Immune-related gene expression study

#### Total RNA extraction

Roughly 50–100 mg of HK tissues was dissected in each fish for isolation of total RNA using TRI reagent (Sigma), per manufacturer’s instruction. The total RNA concentration was determined using spectrophotometer (Nanodrop ND-1000, Thermo Scientific, USA). The sample’s purity was achieved by quantifying the ratio of OD 260 nm/OD 280 nm (1.8–2.0). The purified RNA was then used for cDNA synthesis.

#### Expression study

Two µg of total RNA, which utilized the first strand of cDNA synthesis, were obtained via an Enhanced Avian HS RT-PCR kit (Sigma) through the use of a thermocycler (Mycycler Thermal cycler, BioRad, USA). RNA was kept for 5 min at 80 °C, containing 1 µL of 2.5 µM random hexamer, then kept another 5 min at 4 °C, allowing the primers to anneal of the RNA. A mixture of 10 × MMLV-RT buffer (2 µL), 0.4 U/µL of RNase inhibitor (1 µL), 10 mM dNTPs (1 µL), DEPC water (5 µL), and RT enzyme (1.0 µL) was softly agitated, and kept for 1 h at 42 °C, followed by 10 min at 70 °C. The resulting cDNA was synthesized and kept at 4 °C for further analyses. The constitutive expression of β-actin housekeeping gene was used in both positive control and experimental sample for normalization. The size of cloned PCR products and the primer sequences used for β-actin, as well as the immune-related genes, were investigated. The PCR mixtures contained 2 µL of 10 × PCR buffer, 13.1 µL of dH_2_O, 1.5 µL of MgCl_2_, 0.5 µL of 10 mM dNTPs, 0.5 µL of 0.05 U/µL DNA polymerase (JumpStart Accu-Taq la), 0.2 µL of 10 pmol of both primers (forward and reverse), and 2 µL cDNA. The extension parameters of the PCR as followed: 95 °C for 3 min; followed by denaturation for 30 cycles for 45 s (94 °C), and 45 s for appropriate annealing temperature (Table 2), followed by additional 45 s extension at 72 °C, and a 10-min for final extension (72 °C). The synthesized PCR products were confirmed by 1.0% agarose gel electrophoresis. The relative expression profile of β-actin and immune-relevant genes were evaluated by densitometry (Gel DOC, BIO RAD Laboratories, India).

**Table 2:** List of gene primers used for tissue specific expression pattern of selected immune related genes in *C. catla*.

Primers no	Sequence (5′ → 3′)	Annealing temperature (°C)	PCR amplicon size (bp)	Accession
β-actin	F: ACCCACACTGTGCCCATCTACG	60	146	JQ991014
	R: ATTTCCCTCTCGGCTGTGGTGG			
IL-1β	F: ACCCCACAAAACATCGGCCAACC	60	156	AM932525
	R: TCTTCTCCATTTCCACCCTCTC			
IL-10	F: CGCAGTGCAGAAGAGTCGAC	64	310	GU256643
	R: CCCGCTTGAGATCCTGAAATAT			
IFN-γ	F: AAGGGTTCCTGCTCTTGTCA	54	210	KF590042
	R: GCCATTTTTCACCTCGACTG			
MHC-I	F:AGGAGATGCCGAATGGAG	56	256	MG859931
	R: GATGATTCCCAGCACCAG			
MyD88	F: CTTCCAGTTTGTGCATGAGA	51	146	JN247432
	R: CCATCCTCTTGCACCTTTTT			
NF-kB	F: TTTACAGGAGCGGCGGATAC	59	453	KY089040
	R: GTGCGAAACACGATAGCCAC			
TNF-α	F: CCAGGCTTTCACTTCAGG	56	181	FN543477
	R: GCCATAGGAATCGGAGTAG			
TRAF6	F: CAGTTGACAATGAGGTGCTG	53	328	MF766465
	R: CACACTGTATTGGCGAAAGG			
TLR2	F: GACGGTCATGGATGGTTCTTCTTTA	58	131	HQ293022
	R: CAAGATTGCGTATGTAGGCCGTATG			
TLR3	F: GCTCCACAGGGTTGAAGACA	53	310	MF766464
	R: GCACGGCCAAGCTTTAGAAT			
TLR4	F: ATGATGGAGCGCAATGCCAA	55	140	GU248418
	R: ATGTTACTCAAAGGGTCTCTGCTCC			
TLR5	F: CAGGGTAAACATTTCACGCTTCT	58	162	GU230763
	R: ACGCTTTGCCATGGGAACTTT			
LYZ-C	F: GCTGTGATGTTGTTCGTATCTTC	66	343	JQ230329
	R: GACAGCTTACGCCCATTACAG			
LYZ-G	F: CATGGGACAGTGAGGAACATC	66	204	JQ230328
	R: CATGTGCTCATATGTACGGACG			
NOD1	F: GTTGGTGGGAAATACCTTGCC	56	217	KC542884
	R: TGCTTTCGCCAGACTTCTTCC			
NOD2	F: GGCGGGACAGGACGTTTCTCC	60	261	KC542885
	R: GCGGCAACTGAAGGGGAATA			

*IL-1β/10* interleukins1β/10, *IFN-γ* interferon-gamma, *TNF-α* tumor necrosis factor-alpha, *TRAF6* TNF receptor-associated factor 6, *TLR2/3/4/5* toll-like receptors 2/3/4/5, *LYZ-C/G* lysozyme-C/G, *NOD1/2* nucleotide binding oligomerization domain containing 1/2, *NF-kB* nuclear factor kappa-light-chain-enhancer of activated B cells, *MyD88* myeloid differentiation primary response 88, *MHC-I* major histocompatibility complex-class I.

### Relative percentage survival (RPS)

Analysis of RPS was studied in all the experimental groups as mentioned in “Experimental setup”. Each experimental group was maintained triplicate, and 20 fish were used in each group. The bacterial culture and concentration of bacterial density were the same as mentioned in “Aeromonas hydrophila”. The bacterial challenge and administration of pathogen were the same as mentioned in “Experimental setup”. The survival rate was determined at the end of the experiment. Relative percentage survival (RPS) was calculated following standard formula:

$${\text{RPS}} = 1 - \left[ {\left( {\% \;{\text{mortality}}\;{\text{in}}\;{\text{treatment}}\;{\text{group}}} \right)/\left( {\% \;{\text{mortality}}\;{\text{in}}\;{\text{control}}\;{\text{group}}} \right)} \right] \times 100.$$

### Statistical analysis

Data of each blood and tissue sample were computed as mean ± SEM in triplicate. The percentages of β-actin and the immune gene amplifying products, were later determined and further examined through one-way analysis of variance (ANOVA). Differences were subsequently calculated between the treated groups using Duncan’s Multiple Range (DMR) test, in which significance was considered at *P* < 0.05.

### Ethical approval

The present study follows institutional guidelines mandatory for human and animal treatment and complies with relevant legislation ethical approval from the institute for conducting the research. The animal ethical committee (approval no. 791/03/b/CPCSEA) was approved by the Tamil University, Faculty of Sciences, Department of Siddha Medicine, C-4 Quarters, Thanjavur, 613 005 Tamil Nadu.
